# Deep network-guided key map generation for lossless image encryption

**DOI:** 10.3389/frai.2026.1913975

**Published:** 2026-10-14

**Authors:** C. Mahapriya, V. Thanikaiselvan, Shweta B. Thomas

**Affiliations:** School of Electronics Engineering, Vellore Institute of Technology, Vellore, India

**Keywords:** chaotic map, confusion, deep network, diffusion, image encryption

## Abstract

In this article, a novel 4D Non-monotonic Sinusoidal Chaotic Map (NSCM) for confusion and a deep network-guided key map generation for diffusion are introduced to improve information security against various attacks. This article utilizes the advantages of the sinusoidal function to modify the monotonic function for generating random sequences to obtain a chaotic map for permutation. The limitations of substitution, such as chosen/known plaintext attacks, are suppressed using deep network-based key map generation. A key map equal in size to the size of the plaintext image is obtained as a generative output from a deep network using a smaller map and normalized to the intensity range of the plaintext image. The plaintext image is scrambled block-wise, followed by pixel-wise confusion using the chaotic map. Then the scrambled image is diffused with the key map using a bit-level XOR operation to obtain the encrypted image. The proposed method can be reversed for retrieving the plaintext image, that is, diffusion followed by confusion. The experimental results show that the proposed method performs better qualitatively in terms of visual perception as well as quantitatively in terms of entropy and Peak Signal-to-Noise Ratio (PSNR). Also, the proposed method strongly restricts various attacks such as chosen-plaintext attacks and differential attacks.

## 1 Introduction

This article focuses on encrypting image information by permutation and substitution (Fridrich, 1998; Chen et al., 2004; Preishuber et al., 2018). Permutation performs pixel shuffling, whereas substitution changes the pixel values. In the literature, there have been two major approaches to image scrambling: (i) chaotic map-based confusion (Teng et al., 2025; Gong et al., 2024) and (ii) non-chaotic map-based confusion (Luan and Quan, 2026). Generally, chaotic map-based encryption systems use mathematical functions that are sensitive to initial values to generate random sequences. The initial value is the key in an encryption system, and its strength against various attacks is challenging for securing the information. The non-chaotic map-based encryption system follows predefined rules for changing the positions of pixels of the plaintext image. But confusion is not enough to resist attacks (Jolfaei et al., 2016), and hence researchers utilized diffusion to change the values of pixels. Their permutation techniques were applied at the pixel level, while the substitutions were implemented using modular addition (Chen et al., 2021a). The encryption systems based on permutation and substitution (Chen et al., 2021b) can be simply modeled as:


$$\begin{array}{l} E=M_{i}\left(P\right)⊕K \end{array}$$ (1)

$$\begin{array}{l} M_{i}=V_{i}\left(S_{r}^{i}\right) \end{array}$$ (2)

where is the encrypted image, _*i*_ is the chaotic map obtained from the corresponding index (*V*_*i*_) of the permuted random sequence, $S_{r}^{i}$, *i* represents the number of maps (dimension) and the corresponding number of random sequences (*S*_*r*_), is the plaintext image, is the key map, and the operator ⊕ represents the logical XOR operation. The operation *V*_*i*_(.) represents the permutation in ascending order and _*i*_(.) represents the shuffling of image pixels. The limitations of the above encryption system (Equation 1) are the known/chosen-plaintext attack and differential attacks.

The chaotic map-based confusion methods include Sine Infinite Chaotic Map (SICM) (Zheng et al., 2025), Sine cubic Chaotic Map (SCM) (Li and Teng, 2025), Inserted Logistic-Logarithmic-Sine Chaotic Map (ILLSCM) (Liu et al., 2025), and Non-linear Sine-Log-Sqrt-Logistic Map (2D-NSLSLM) chaotic map (Wang et al., 2025). A 3D cat map was used for confusion, and a 3D logistic map (Qian et al., 2021) was used for diffusion to encrypt color images. A composite encryption system (Song et al., 2021) was introduced in which sine, logistic, and generalized third-order Fibonacci were cascaded, followed by bit scrambling. A dynamic diffusion algorithm (Wang N. et al., 2019) was proposed by hybridizing two chaotic systems—logistic and logistic-adjusted sine maps for image encryption. A cloud model Fibonacci chaotic method (Hu et al., 2020) was proposed for encryption of RGB color images. A different approach was proposed for image compression and encryption using tent-sine, Arnold, and Chen maps using compressive sensing and Fourier transform (Zhang et al., 2020).

The non-chaotic map-based encryption systems have the limitation of being prone to chosen/known-plaintext attacks since there is a possibility for intruders to know the predefined rules. On the other hand, as the key image also plays a vital role in encryption, many researchers have focused on it. So, the generation of random sequences to obtain the chaotic maps and the synthesis of the key image need to be focused on to secure the image information (Chen et al., 2021b). Some researchers followed row-column shuffling for scrambling, followed by diffusion (Anujaa et al., 2025; Sivaranjani Devi and Amirtharajan, 2025). To avoid the limitation of degradation, which commonly occurs in chaotic systems, a hyperchaotic system, which was a Non-Degenerate Hyperchaotic System (4D-NDS), has been introduced for confusion, followed by permutation, bit-wise diffusion, row-column diffusion, and XOR confusion (Liao et al., 2025). The permutation was performed by a circular shift of the pixel level using a pseudo-random generator (Diaconu, 2016). A 2D chaotic map was generated using SHA-256 for key generation, wherein permutation was performed by row and column shifting using the first chaotic map, and substitution was performed in a stream of bytes using the second chaotic map (Moysis et al., 2025), Arnold transform-based confusion, followed by three levels of diffusion, such as the particle swarm optimization-based S-box diffusion, pixel-level diffusion, and heterogeneous diffusion, was used for image encryption (Song and Zhao, 2025). A Discrete Wavelet Transform (2D-DWT)-based phase shift technique was also used for encryption (Xiao and Liu, 2025). The color images were encrypted in three stages (Abduljabbar et al., 2022), including swapping bits of each pixel followed by scrambling using the Zaslavsky and Hanon map, and XOR diffusion using a key image generated by HyperLogVarHanon. SHA-256-based key generation for a piecewise linear chaotic system (Sun and Chen, 2022) was also proposed for image encryption.

Further, the researchers used DNA coding (Wang and Lai, 2025; Zhang et al., 2024) for diffusion, which improves the robustness of the encryption system against various attacks. The one-dimensional Chebyshev and Sine maps were extended for chaotic jump and parallel encoding using the DNA algorithm for image encryption (Zhang and Ma, 2023). Another color image encryption approach was introduced by scrambling with the Arnold map followed by DNA coding and diffusion with Lorentz and Rössler maps (Liu and Liu, 2020). However, the time required to complete the overall encryption process using DNA coding is longer during implementation.

The applications of image encryption have been extended to include medical images, including a hyperchaotic system framed with a quantum cellular neural network and game theory (Zhou et al., 2020) for optimized lossless encryption of the region of interest in medical images. The encryption of medical images is performed by scrambling image blocks followed by diffusion using an XOR operation with a key image generated by logistic sequences (Kamal et al., 2021). The amalgamation of many cryptographic techniques to encrypt medical images was introduced in Ningthoukhongjam et al. (2024). The medical images with tumors were encrypted by scrambling followed by forward and reverse diffusions (Wang X. et al., 2019). A hyperchaotic system was proposed based on the Fibonacci Q-Matrix, Galois Field, and memristive coupled neural network for encryption of medical color images (El-Damak et al., 2024). Image compression and encryption were performed for secure transmission of images in the Internet of Medical Things (Lai and Ji, 2025) based on bit plane slicing followed by encryption using a 4D hyperchaotic map. The technique of reversible data hiding was used to embed the patient's privacy data in medical images, followed by encryption (Yang et al., 2020). UNet3+ was used to segment the region of interest (Singh K. N. et al., 2024) in the medical images, and the hybrid chaotic map was utilized for encryption using the features obtained from iris and fingerprint to ensure strong security for medical images. A 2D compressive technique was used to compress the medical images, followed by a 7D hyperchaotic map (Kaur et al., 2023) for encryption to secure the medical images. Medical images were encrypted by two modules (Wu et al., 2026), such as a large language model, which improved the DNA coding and content-aware permutation and diffusion.

Recently, researchers have extended the applications of deep learning to image encryption, including improved GANs (Singh M. et al., 2024) and feature fusion encoding (Long et al., 2024). A hiding network was framed based on SegNet for double encryption (Hualong and Daidou, 2024). Further, a critical comparison of the recent encryption methods is shown in Table 1.

**Table 1:** Critical comparison of recent encryption methods.

References	Significance	Techniques	Image type	Limitations
Liao et al. (2025)	4D-NDS	Initial values *x* with four sub-values *x*_1_(1) = 0.1, *x*_2_(1) = 0.2, *x*_3_(1) = 0.3 and *x*_4_(1) = 0.4, σ(*n*) = 1, ε(*n*) = 1, and SHA-256.	Grayscale image	Vulnerable to attack
Raghuvanshi et al. (2024)	Intertwining Logistic +DNA+VGG16	Sixteen subkeys from VGG16 to generate SHA-512 for the public key, and a pseudorandom number generator to derive the private key to obtain the main key for intertwining the logistic map for confusion. DNA coding for diffusion.	Color images, black-and-white images	Time complexity is high
Yogi et al. (2025)	Combination of the Tinkerbell and tent maps	Combined Tinkerbell-Tent Maps and piecewise linear chaotic map having initial values *x*_0_, *y*_0_, constants “*a*” and “*r*” and blending factor α.	Grayscale image	Vulnerability to chosen-plaintext attack
Safdar et al. (2025)	S-Box + DNA	Chain ring defined using a Galois Field with parameters “*a*, ” “*r*, ” and “*n*” for S-box generation and DNA coding-based diffusion.	Multiple color images	Limited adaptability and vulnerability to chosen-plaintext attack
Li and Tang (2025)	2D Quantum	Quantum number-based reversible color-to-grayscale conversion techniques with a 32-bit hash key and a 32-bit custom key	Color and grayscale	Vulnerability to chosen-plaintext attack
Song and Zhao (2025)	Integrated maps	Hyperchaotic Chen with parameters *m* = 35, *n* = 3, *p* = 12, *q* = 7, *r* = 0.798, Lorenz map with parameters σ = 10, ρ = 28, β = 8/3 and Hénon map with parameters *a* = 1.4, *b* = 0.3	Grayscale image	Vulnerability to chosen-plaintext attack
Alawida and Teh (2026)	3D S-Box	A secret key of 256 bits for the generation of sixteen 3D S-boxes	Color images of different sizes	Key space is low

The known/chosen-plaintext attack is a threat to encryption systems that work on either confusion or diffusion. If the systems use both confusion and diffusion for image encryption, then the strength is greater. In order to make the system more secure against various attacks, the derivation of is very crucial. In the literature, many researchers used either the same random sequences that they used for confusion to derive or used a different function to generate a random sequence to derive. As the usage of _*i*_ and in image encryption enhances the strength of the cryptosystem, this article introduces a new random sequence generator by utilizing the advantages of the sinusoidal function to modify the monotonically increasing sequence generating function and termed the Non-monotonic Sinusoidal Chaotic Map (NSCM). Also, a new approach is proposed to generate to ensure more complexity against attacks. The proposed derivation of is given . by:


$$K_{m\times n}=mod\;(CL_{m\times n}^{TC}\left(reshape\left(S_{r}^{1\times 16}\left(4\times 4\right)\right)\right),\;L-1)$$ (3)

where *m*×*n* is the dimension of which is the same as the size of *CL*^*TC*^(.) represents the operation of a Convolution Layer with transposed convolution with input size 1 × 16 and *L* is the maximum quantized pixel level of (usually 256 for 8-bit images).

The main contributions of this article are:

1. A new 4D chaotic system is introduced for image scrambling.

2. Deep network-guided key map generation is introduced for diffusion.

The novelty and main significance of the proposed system are the generation of a highly random sequence by converting a non-random monotonically increasing function into a random non-monotonic function. Also, the key map, of size equal to the size of the plaintext image, is generated from the layers of a Convolutional Neural Network with transposed convolution, with an input vector of size 4 × 4 which is also a key for the encryption system. The generation of the random sequence depends on the keys (initial values and control parameters), and the synthesis of the key map depends on the random sequences to obtain the input vector of size 4 × 4 from any of the four sequences, having a total number of elements equal to the total number of pixels of the plaintext image. Hence, the key complexity of the system is increased, and it provides high security for the information. Instead of converting the sequences into a key image for diffusion, this method chooses only 16 elements and utilizes the advantages of CNN with transposed convolution to get the key image. As the proposed CNN does not require training and testing, the time complexity is also not a concern. The network layer and its parameters are not fixed, and they can be varied according to user needs and are known only to the sender and receiver. Thus, this can also be treated as part of the secret key for further enhancement of the key complexity.

The rest of the study is organized as follows. Section 2 deals with Preliminaries, Section 3 describes the Proposed methodology, Section 4 demonstrates the Results and discussion, Section 5 demonstrates Comparative analysis, and the Conclusion is in Section 6.

## 2 Preliminaries

### 2.1 Random sequences

The monotonic function generates a sequence of numbers in ascending or descending order (Kaczor and Nowak, 2009). If the numbers are in ascending order, then the function is called an increasing function; otherwise, it is a decreasing function. The mathematical expression of the monotonic function is given by Equation 4 as:


$$\begin{array}{l} a\left(n+1\right)=a\left(n\right)+\left(1/n\right)-\left(n+1\right)/n\\ b\left(n+1\right)=a\left(n\right)+\left(1/n+1\right)-\left(n+1\right)/n \end{array}$$ (4)

where *a*(*n*) and *b*(*n*) represent the value of present elements, *a*(*n*+1) and *b*(*n*+1) represent the value of the next elements, and *n* refers to iterations.

The sequences generated by the monotonic function are non-random. To obtain a chaotic map from the sequences, only the random sequences are required. Generally, the sinusoidal function is used to generate a complex, unpredictable sequence (Zou et al., 2023) that enhances chaos, as it is sensitive to initial values and has a large key space. To extend the two sequences (non-random) generated by Equation 4 into four sequences (random), it is modified by utilizing the advantages of the sinusoidal function. In this way, the key space of the sinusoidal function is further expanded, and non-random (ascending or descending) sequences of the monotonic function are converted into random sequences. To the best of our knowledge, this article is the first in the literature to propose a new strategy for generating random sequences by modifying a non-random function for image encryption. The proposed modification of the monotonic function in Equation 4 is given by Equation 5 as:


$$\begin{array}{l} a\left(n+1\right)=b\left(n\right)-\left(m\times \sin\left(a\left(n\right)\right)\right)\\ b\left(n+1\right)=a\left(n\right)+sin\left(1/\left(n+1\right)\right)-\left(n+1\right)/n\\ \;c\left(n+1\right)=m\times a\left(n\right)\times \sin\left(1-b\left(n\right)\right)\\ d\left(n+1\right)=a\left(n\right)+b\left(n\right)+c\left(n\right) \end{array}$$ (5)

where *m* is the control parameter of the proposed NSF. The initial values should lie in the range of 0 to 1 with 15 decimal places, and the control parameter value should lie between 3.4 and 4 with 15 decimal places. After generating the random sequences using the proposed Equation 5, from four different sequences, each having 65,536 elements (if the total number of pixels of the plaintext image is 65,536), only 16 elements are chosen and converted into a vector of size 4 × 4. To implement the proposed approach, the initial values and control parameters of NSF are set as: *a* = 0.01, *b* = 0.5650, *c* = 0.5007, and *m* = 3.8691 to generate the random sequences. As the dimension of the plaintext image is considered *u*×*v* and *u* = *v* = 256, using Equation 5, four random sequences are generated, each containing a total of *u*×*v* elements, and in vector representation it is 1 × (*u*×*v*). As Equation 5 is a function, it requires initial values of the variable “*n*” for the first three sequences, that is, *a*(0), *b*(0), and *c*(0) to generate the random sequences.

### 2.2 Analysis of Lyapunov exponent and bifurcation

The chaotic behavior of a dynamic system is analyzed using the Lyapunov exponent (λ) and bifurcation analysis, which reflect the sensitivity to the initial values and the control parameter (*m*). In general, chaotic behavior is high when λ is positive (Li et al., 2025; Huang and Gao, 2024; Ying-Qian et al., 2018). The sequences *a*(*n*) and *c*(*n*) of the proposed 4D NSF produce the positive values of λ_*a*_ = 0.85 and λ_*c*_ = 0.69 at *m* > 3.6 and *m* > 3.7, respectively. In the bifurcation diagram, the periodic window occurs at the bifurcation points whenever the value of*m* reaches 1.04 and 3.99 for *a*(*n*) with full bifurcation from 2.26, and for *c*(*n*), it occurs when *m* reaches 2.99 and 3.99, with full bifurcation from 3.54. This shows that the doubling of the periodic window occurs up to 3.54, where the map becomes chaotic, and at 3.99, the system becomes more chaotic. Figure 1 shows the Lyapunov exponent and bifurcation diagram of the sequences *a*(*n*) and *c*(*n*) of Equation 5 with respect to *m*. In Figure 1A, the horizontal axis represents the value of the control parameter (*m*), and the vertical axis represents the value of the Lyapunov exponents for *a*(*n*) and *c*(*n*). In Figures 1B, C, the horizontal axis represents the value of the control parameter, and the vertical axis represents the sequences *a*(*n*) and *c*(*n*), respectively. Even though the proposed NSF generates four sequences, the fourth sequence is mainly used to generate a key map for diffusion. As the trajectories of *a*(*n*) and *c*(*n*) diverge for *m* > 3.6 and *m* > 3.7, and produce positive Lyapunov exponents (λ_*a*_ and λ_*c*_ > 0), the system exhibits chaos. Both sequences bifurcated when *m* > 3.5, which also shows that the system is chaotic. Further, for *a*(*n*), the 0–1 Test returns 0.84, which is greater than the transition range, and hence the proposed system exhibits chaos.

**Figure 1:**
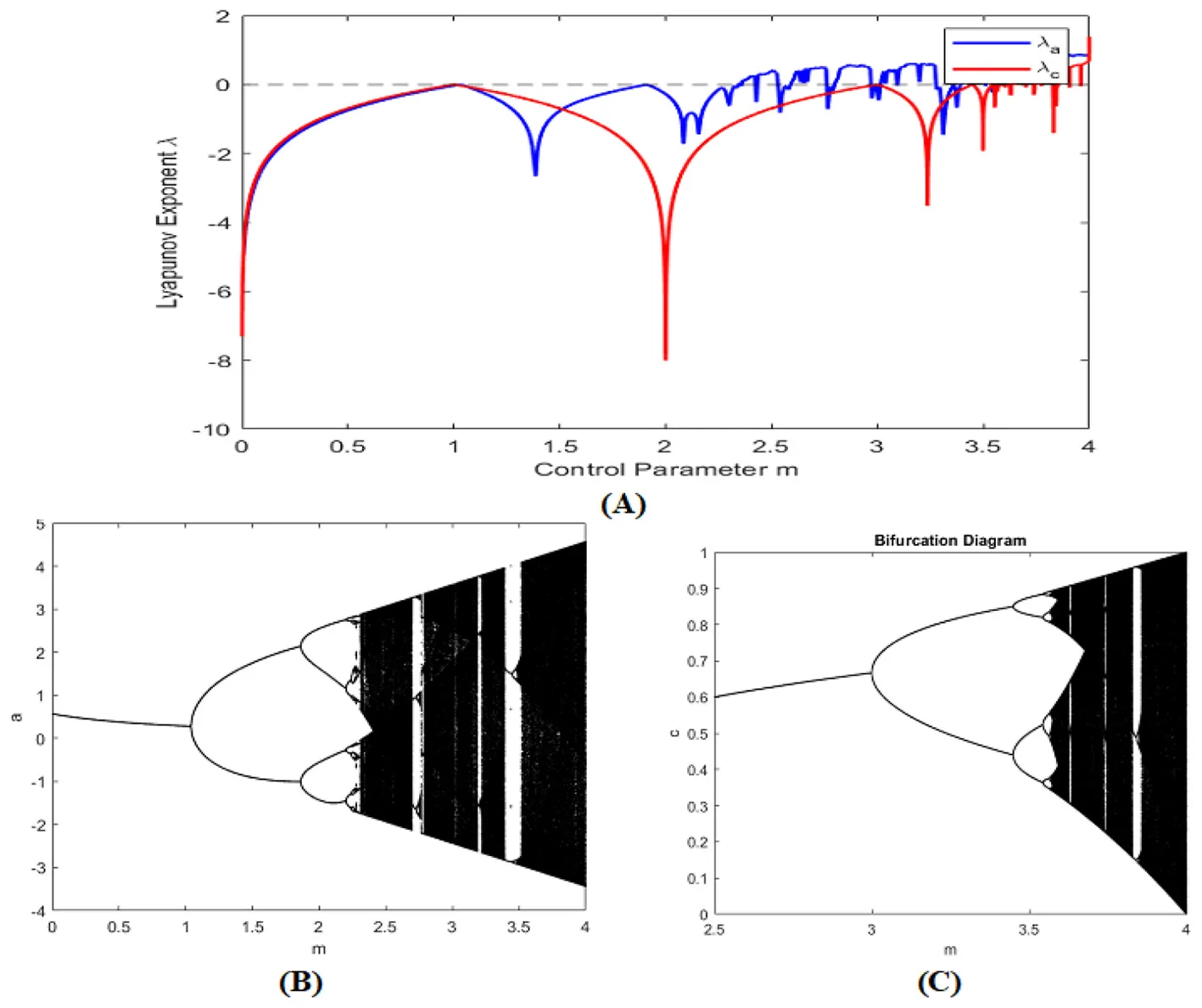
**(A)** Lyapunov exponent for *a*(*n*) and *c*(*n*), **(B)** Bifurcation analysis for *a*(*n*), and **(C)** Bifurcation analysis for *c*(*n*).

### 2.3 NIST randomness test

Usually, the randomness of the binary information (sequence) is tested using tests proposed by NIST (Rukhin et al., 2010). NIST is a statistical analysis used to test the sequence in different ways, such as the run test, the frequency test, etc. The results of the NIST Test for the proposed 4D chaotic map using the NIST suite are depicted in Table 2. Hence, the proposed NSF improves the randomness of the information in the encrypted image.

**Table 2:** NIST performance test.

Test	*p*-value	Pass or not?
Frequency Test (Monobit)	0.0307	Pass
Frequency Test Within a Block	0.6289	Pass
Run Test	0.2165	Pass
Binary Matrix Rank Test	0.9581	Pass
Discrete Fourier Transform	0.6618	Pass
Non-Overlapping Template Match	0.6176	Pass
Overlapping Template Match	0.9999	Pass
Linear Complexity Test	0.6418	Pass
Serial Test	0.0979	Pass
Approximate Entropy Test	0.0419	Pass
Cumulative Sums (Forward) Test	0.0108	Pass
Cumulative Sums (Reverse) Test	0.0469	Pass
Random Excursions Test	0.1812	Pass
Random Excursions Variant Test	0.7561	Pass
Longest Run Test	0.8981	Pass

## 3 Proposed methodology

The workflow of the proposed method is shown in Figure 2, and Figure 3 shows the layers of the deep network for generation of the key map. The plaintext image is confused block-wise, followed by two rounds of pixel-wise confusion. A key map equal in size to the size of the plaintext (typically **256 × 256**) is generated using a deep network with six convolutional layers of transposed convolutional layers using a key map of size **4 × 4** matrix, which is taken from the fourth sequence of 4D NSCM. Then the confused image and the key map are diffused at the bit level using an XOR operation to obtain the encrypted image.

**Figure 2:**
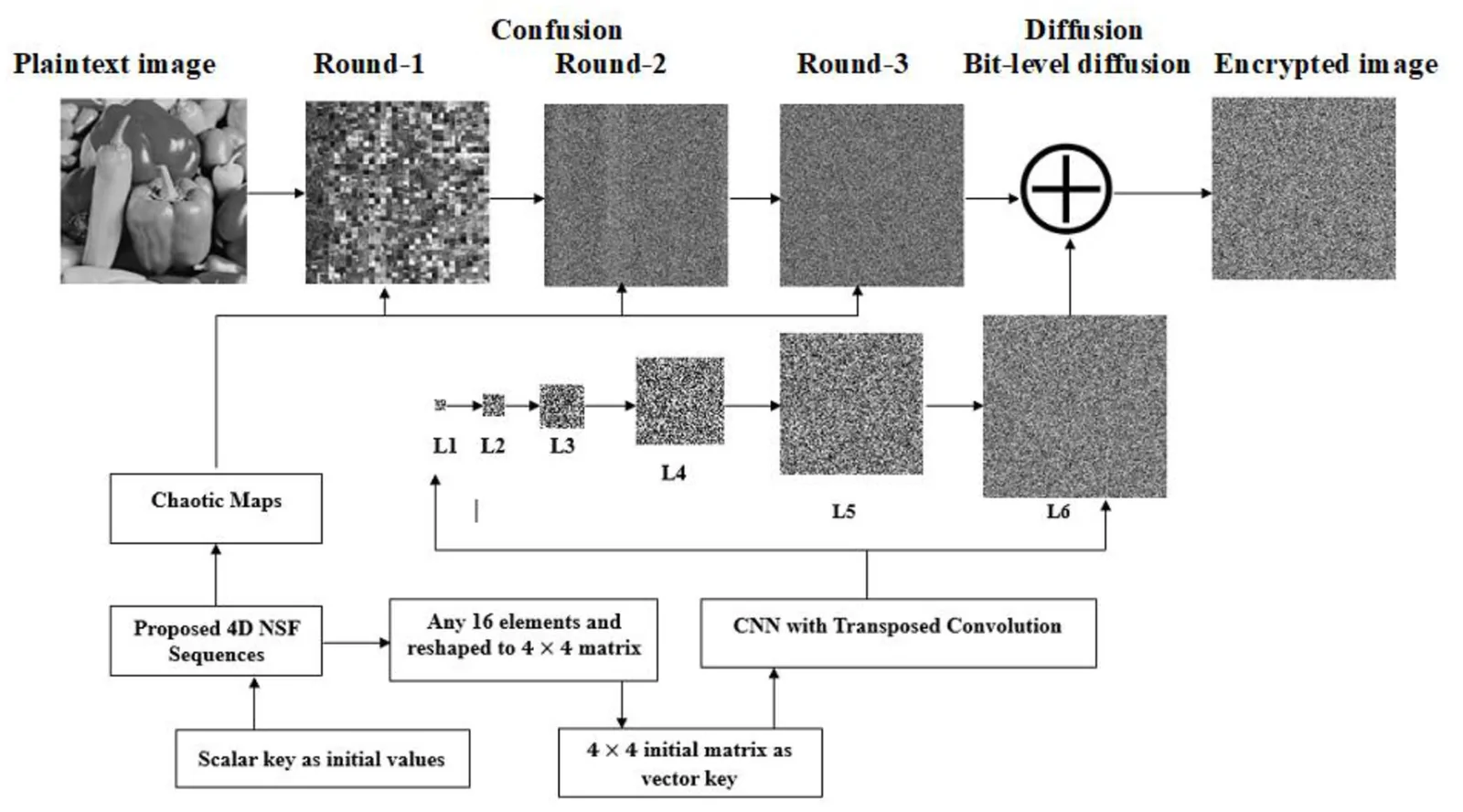
Workflow of the proposed encryption method (plaintext image reproduced from the USC-SIPI Image Database, USC-SIPI Image Database [2025], https://sipi.usc.edu/database/).

**Figure 3:**
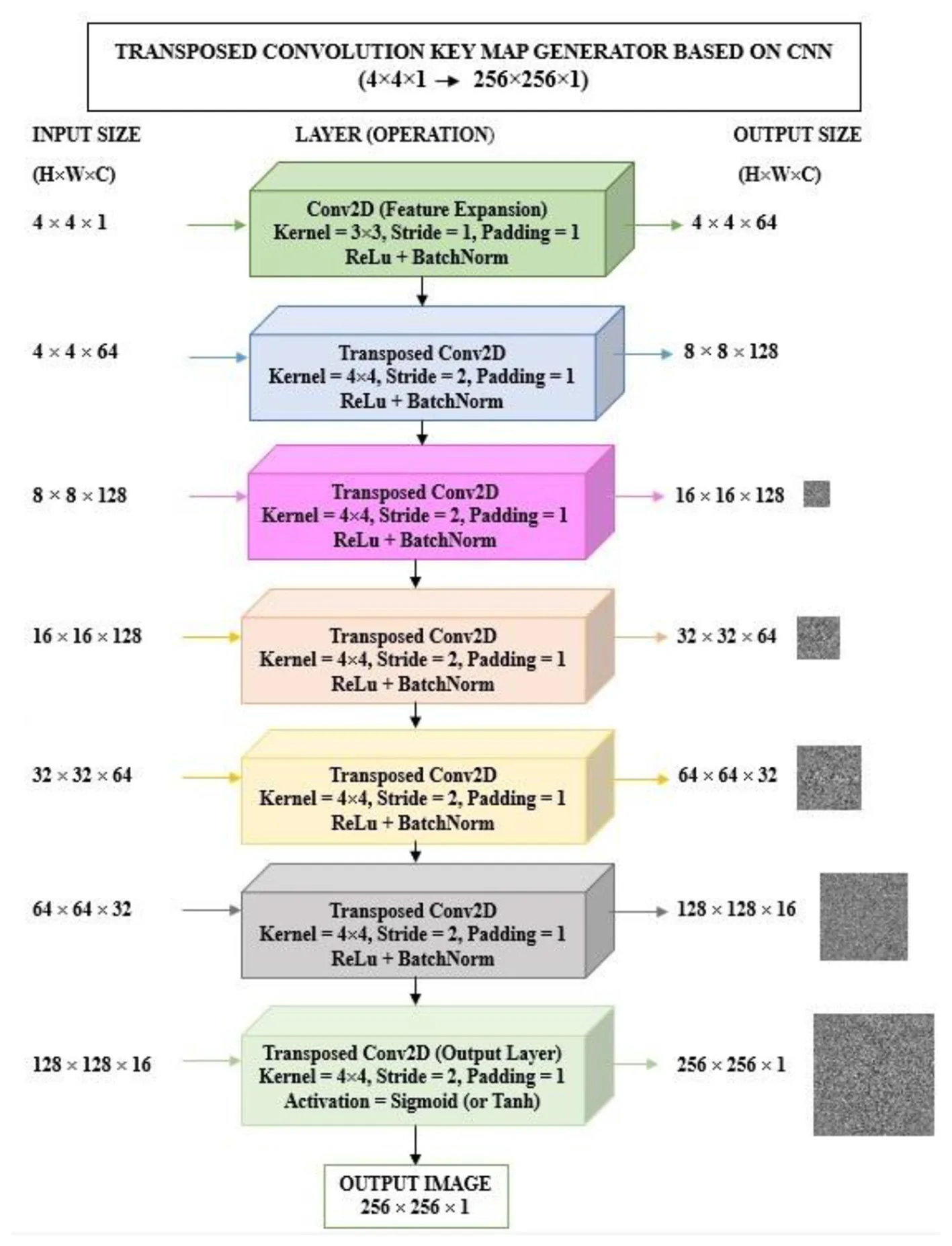
Proposed key map generator. Conv2D represents the 2D Convolution.

### 3.1 A non-monotonic sinusoidal chaotic map

Using the key values for Equation 5, the following four random sequences are generated and are given in Equation 6.


$$\begin{array}{c} \begin{array}{c} A\left(n\right)=\;{\left\{a\left(n+1\right)\right\}}_{n=0}^{n=uv-1}\\ B\left(n\right)=\;{\left\{b\left(n+1\right)\right\}}_{n=0}^{n=uv-1}\\ C\left(n\right)=\;{\left\{c\left(n+1\right)\right\}}_{n=0}^{n=uv-1}\\ D\left(n\right)=\;{\left\{d\left(n+1\right)\right\}}_{n=0}^{n=uv-1} \end{array} \end{array}$$ (6)

Let *A*(*n*), *B*(*n*), and *C*(*n*) represent the sequences of random numbers generated by expanding, respectively *a*(*n*), *b*(*n*), and *c*(*n*) of Equation 6, up to 65,536 iterations. The elements of the random sequences are indexed and sorted in ascending order by their values. The permutation provides a disordered index (*V*_*i*_) which is considered a chaotic map used for shuffling the plaintext image as represented in Equation 2 and expressed by Equation 7:


$$\;\begin{array}{c} A=\left\{a\left(1\right),\;a\left(2\right),\;\ldots ,\;a\left(uv\right)\right\}\\ B=\left\{b\left(1\right),\;b\left(2\right),\;\ldots ,\;b\left(uv\right)\right\}\\ C=\left\{c\left(1\right),\;c\left(2\right),\;\ldots ,\;c\left(uv\right)\right\}\\ D=\left\{\begin{array}{c} a\left(1\right)+b\left(1\right)+c\left(1\right),\;\\ \;a\left(2\right)+b\left(2\right)+c\left(2\right)\ldots ,\;\\ a\left(uv\right)+b\left(uv\right)+c\left(uv\right) \end{array}\right\} \end{array}\}$$ (7)

Then the sequences **A**, **B**, **C**, and **D** in Equation 7 are the expansions of *A*(*n*), *B*(*n*), *C*(*n*), and *D*(*n*) up to *u, v* and are indexed separately to identify the position of the elements of the sequences, and the indexed sequences are represented by Equation 8.


$$\begin{array}{c} A=\left\{\left[1,a\left(1\right)\right],\;\left[2,\;a\left(2\right)\right],\;\ldots ,\left[\left(uv\right),\;a\left(uv\right)\right]\right\}\\ B=\left\{\left[1,\;b\left(1\right)\right],\;\left[2,\;b\left(2\right)\right],\;\ldots ,\left[\left(uv\right),\;b\left(uv\right)\right]\right\}\\ C=\left\{\left[1,\;c\left(1\right)\right],\;\left[2,\;c\left(2\right)\right],\;\ldots ,\left[\left(uv\right),c\left(uv\right)\right]\right\} \end{array}\}$$ (8)

where Ā, $\bar{B}$, and $\bar{C}$ denote the indexed sequences corresponding to **A**, **B**, and **C**, respectively. The indexed sequences of Equation 5 are arranged in two columns such that the first column represents the element and the second column represents the corresponding index. Based on the values of the elements of Ā, $\bar{B},$ and $\bar{C}$, that is, the second row of the indexed sequence is sorted in ascending order to obtain the ordered sequence, but the index becomes disordered. The chaotic index of Ā, $\bar{B}$, and $\bar{C}$ of Equation 7 are considered the chaotic maps *A*, *B*, and *C* each with a dimension of 1 × (*u*×*v*), respectively. The elements of the fourth sequence, *D*(*n*) of Equation 5, that is, **D** are used to generate a key map, which is also dependent on other sequences. There are four chaotic maps derived from Equation 6, and hence it is called 4DNSCM.

### 3.2 Generation of key map

According to Equation 2, the derivation of *K* by the proposed method needs 1 × 16 vector from 4DNSCM. The elements of the fourth sequence, **D** in Equation 6 are in the form of 1 × 65, 536. From the 65,536 values of **D**, 16 elements are randomly chosen and reshaped into 4 × 4. Using a deep network (Glorot and Bengio, 2010; He et al., 2015; Fridrich, 1998) with transposed convolution, 4 × 4 matrix is converted into the key map (*K*) of size 256 × 256. Figure 3 shows the layers of the deep network to generate *K*. The size of the input is increased by three sequential operations, namely, upsampling, non-linearization, and stabilization of the input features (values) in each layer of the deep network. The output of each layer (*O*) of the deep network is represented by Equation 9.


$$\begin{array}{c} O^{l+1}=\sigma \left(k^{l}⊗x^{l}+b^{l}\right) \end{array}$$ (9)

where, *x*^*l*^, *k*^*l*^, and *b*^*l*^ are the input feature values, the kernel of the transposed convolution, and the bias of the layer (*l*), *l* = 0 represents the input layer, ⊗ represents the convolution operation, respectively, and σ(.) represents the activation function used in the deep network. In this study, the transposed convolution upsamples the input size, the rectified linear unit (ReLU) is used as the activation function for learning complex patterns to make the inputs non-linear, and the stabilization of inputs is performed by batch normalization. The ReLU is mathematically represented by Equation 10.


$$\begin{array}{c} ReLU=max\left(0,\;x\right) \end{array}$$ (10)

That is, ReLU allows only the positive values of *x* otherwise; it considers only 0 for negative values of *x*. Thus, the output of ReLU is non-linear. The batch normalization technique (He et al., 2015) is used to stabilize the non-linear values of ReLU.

The size of the output of each layer in a deep network is represented by Equation 11.


$$\begin{array}{c} o=\left(x-1\right)s-2p+K+q \end{array}$$ (11)

where *o, x, s, p, q*, and *K* are the size of the output, input, stride, padding, output padding, and kernel, respectively, for each layer. The input layer of the deep network is given as follows.


$$\begin{array}{c} x^{0}\in {\mathbb{R}}^{4\times 4} \end{array}$$

To obtain the output size of 256 × 256 from the input size of 4 × 4, *K* = 4, *p* = 1, *q* = 0, and *s* = 1 for the first layer and for other layers, *s* = 2 to double the size of the input. Therefore, the output of the first through the sixth layer of the deep network is given by:


$$\begin{array}{c} O^{1}=\sigma \left(k^{1}⊗x^{0}+b^{1}\right)\\ O^{2}=\sigma \left(k^{2}⊗x^{1}+b^{2}\right)\\ O^{3}=\sigma \left(k^{3}⊗x^{2}+b^{3}\right)\\ O^{4}=\sigma \left(k^{4}⊗x^{3}+b^{4}\right)\\ O^{5}=\sigma \left(k^{5}⊗x^{4}+b^{5}\right)\\ O^{6}=\sigma \left(k^{6}⊗x^{5}+b^{6}\right) \end{array}$$

where *x*^1^ = *O*^1^, *x*^2^ = *O*^2^, *x*^3^ = *O*^3^, *x*^4^ = *O*^4^, and *x*^5^ = *O*^5^. The size of the input, the output of each layer, the kernel size, and the number of kernels of the deep network are given in Table 3. Each layer of the deep network gradually increases the size of the input image to 4 × 4 to 256 × 256. The deep network upsamples the size of the input layers using filters and kernel size to generate high-resolution output.

**Table 3:** Output size of each layer of the deep network.

Layer	Input size	Kernel size	No. of filters	Output size
0	4 × 4	–	–	–
1	4 × 4	3 × 3	64	8 × 8
2	8 × 8	4 × 4	128	16 × 16
3	16 × 16	4 × 4	128	32 × 32
4	32 × 32	4 × 4	64	64 × 64
5	64 × 64	4 × 4	32	128 × 128
6	128 × 128	4 × 4	16	256 × 256

The size of the initial 4 × 4 vector is chosen for the experimental purpose. The determination of the size of the initial vector is done by the user, and also, the proposed CNN layers have been designed accordingly. Hence, this type of synthesis of the key map is not fixed and provides high security to the encrypted image during diffusion. Synthesizing the key map of size 256 × 256 from a smaller size of 4 × 4 instead of 128 × 128, 64 × 64 may reduce the key complexity. So, it is better to choose a lower-sized initial value to generate a higher value using transposed convolution.

The training and testing of the proposed CNN are not separate encryption stages. As the proposed deep network is not used for classification, regression, or feature learning, it does not require training and testing, and its parameters are predetermined and fixed, which may serve as keys. The proposed CNN-based deep network does not require backpropagation, and it works only in the forward direction up to a predetermined number of layers to expand the 4 × 4 input vector into a key map of size 256 × 256. So, it is employed as a deterministic non-linear mapping through fixed transposed-convolution layers for key map synthesis.

The size of the input can be increased or decreased using convolutional neural networks, and this approach is used in this study. The proposed CNN is used only for the synthesis of a key map the same in size to the size as the plaintext image from a smaller vector (preferably 4 × 4) with the concept of transposed convolution over six stages. The concept of interpolation for upsampling has the limitation of vulnerability to known-plaintext attacks. Other methods, such as pseudo-random key expansion methods, have many limitations, such as time complexity and vulnerability to attacks. To overcome the disadvantages of the proposed method, the selection or determination of the input 4 × 4 vector depends on the random sequences. Also, the values of each element in the vector can be varied up to 15 decimal places. Hence, the key complexity and key sensitivity have been increased in the proposed method.

As the elements of the output of the 6th layer of the deep network have low values, they are normalized to the range [0, 255]. The normalized sequence *K* is reshaped from 1 × 65, 536 to 256 × 256 to generate a key map (*K*) as shown in Equation 3. The generated key map is shown in Figure 4. Since there is no correlation between pixels of the key map, it appears like a scrambled image.

**Figure 4:**
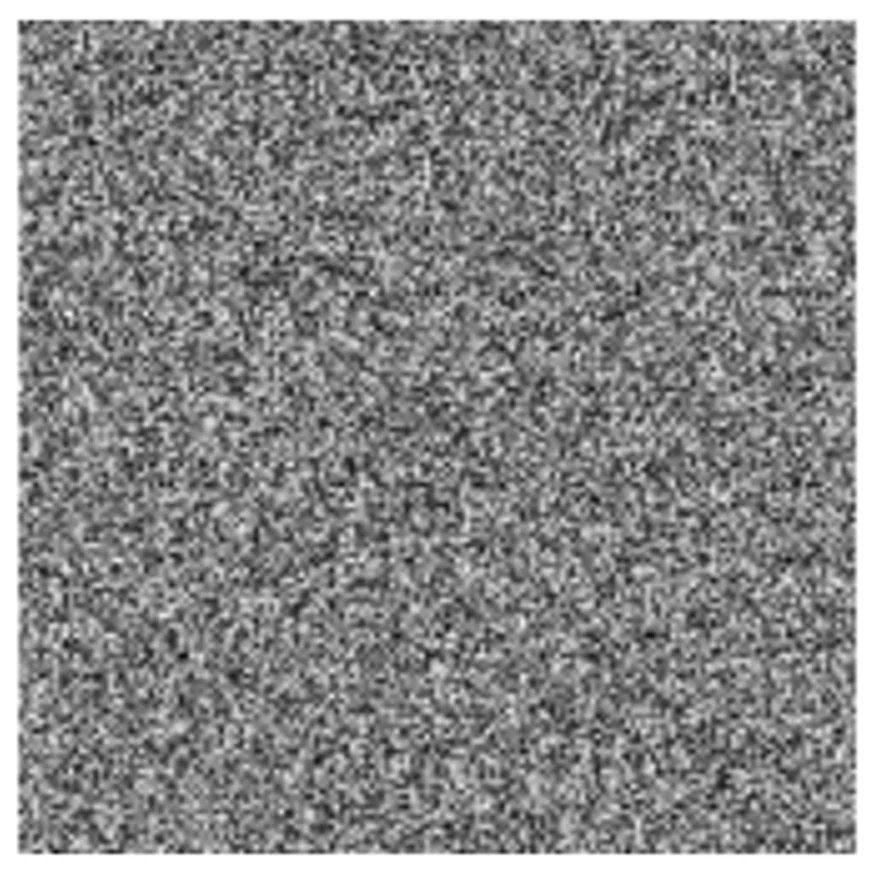
Generation of the key map (256 × 256) using a deep network.

This study is implemented using the images represented by unsigned 8-bit integers. The size of the generated key map is equal to the size of the plaintext image, that is, 256 × 256. Therefore, the key generation consumes a total of 64 KB, 64 KB, and 192 KB of memory, respectively, for a grayscale image, key map, and color image in the RGB plane.

### 3.3 Confusion

Confusion is performed by scrambling the plaintext image by permuting pixel positions, whereas diffusion changes the intensity value of all pixels. In general, complexity is higher in diffusion than in confusion. So, this study proposes one round of image block confusion and two rounds of image pixel confusion. The rationale behind the three rounds of confusion is to increase the complexity. The first round of confusion is achieved image-block-wise through row-scrambling the image blocks using the chaotic map derived from the first sequence of 4D-NSF. The second and third rounds of confusion are achieved pixel-wise using the chaotic maps derived from the second and third sequences, respectively, of 4D-NSF. In this way, the three rounds of confusion make the plaintext image more indecipherable, and the process is also more complex against various attacks. Therefore, Equation 5 has advantages over Equation 4 because of image-block-wise confusion and pixel-wise confusion in three rounds.

#### 3.3.1 Image-block-wise confusion

As the dimension of the plaintext image (*I*) is considered *u*×*v* and *u* = *v* = 256, the total number of pixels is *u*×*v* = 65, 536 and is represented by:


$$\begin{array}{c} I=\left\{I\left(u,\;v\right),\;1\leq u,\;v\leq 256\right\} \end{array}$$

where *I*(*u, v*) is the pixel intensity at location (*u, v*). *I*(*u, v*) is divided into image patches/blocks (*f*) of each size *m*×*n* where *m* = *n* = 8. The dimension of the image, *I* in terms of image blocks is *M*×*N* where *M* = *N* = 32. The total number of image blocks, *n* represented by Equation 12.


$$\begin{array}{c} M=n_{H}=\frac{u\;\left(=256\right)}{m\left(=8\right)}=32 \end{array}$$

$$\begin{array}{c} N=n_{W}=\frac{v\;\left(=256\right)}{n\left(=8\right)}=32 \end{array}$$ (12)

The total number of blocks is:


$$\begin{array}{c} n=n_{H}\times n_{W} \end{array}$$

Therefore, 1,024 image blocks of size 8 × 8 each is obtained. Each image block (*f*) is represented as an element of the image, *I* is $I_{M,\;N}\in {\mathbb{R}}^{8\times 8}$, where:

*M* = 1, 2, 3, …, 32, and *N* = 1, 2, 3, …, 32

Then, the (*M, N*)^*th*^ image block in the plaintext image is represented by Equation 13.


$$\begin{array}{c} I_{M,\;N}=\left[\begin{array}{ccc} I\left(8m-1,\;8n-7\right) & \cdots & I\left(8m-7,\;8n\right)\\ \vdots & \ddots & \vdots \\ I\left(8m,\;8n-7\right) & \cdots & I\left(8m,\;8n\right) \end{array}\right] \end{array}$$ (13)

The image blocks can be reshaped into a column vector as:


$$\begin{array}{c} f_{i}={\mathbb{R}}^{64\times 1} \end{array}$$

where, *i* = 1, 2, 3, 4, …, 1, 024. All image blocks can be represented as a vector of size 1 × 1024 as:


$$\begin{array}{c} F=\left[f_{1},\;f_{2},\;f_{3},\;\ldots ,\;f_{1024}\right] \end{array}$$

Therefore, $I\in {\mathbb{R}}^{256\times 256}={\left\{I_{M,N}\right\}}_{M,N=1}^{32}\to F\in {\mathbb{R}}^{64\times \;1024}$

The sequence *A*(*n*) is generated again up to 1,024 elements and indexed. The indexed sequence is sorted in ascending order, and the disordered index is considered the chaotic map, *A*(*i*). The sub-images are also considered the elements and reshaped into 1 × 1024, indexed and reordered according to *A*(*i*). Therefore, the image-block-wise confusion is represented as:


$$\begin{array}{c} F^{1}=\;F_{A\left(i\right)}\in {\mathbb{R}}^{256\times \;256} \end{array}$$

where *F*^1^ is the block-wise-confused image. The reordered sub-images are reshaped into 32 × 32 blocks. Therefore, the image information is confused and converted into a scrambled image, *F*^1^ which is called a first-round scrambled image. However, the dimension of *F*^1^ is 256 × 256 pixels.

#### 3.3.2 Image pixel-wise confusion

To improve security against various attacks, the block-wise-confused plaintext image is again confused pixel-wise. The first-round scrambled image, *F*^1^ is reshaped from 256 × 256 to 1 × 65536 and indexed and reordered the pixels indexed and reordered in accordance with the chaotic map *B*(*i*) to obtain the second-round scrambled image, *F*^2^.


$$\begin{array}{c} F^{1}\in {\mathbb{R}}^{256\times 256}\to F^{1}\in {\mathbb{R}}^{1\times \;65536}\\ F^{2}=\;F_{B\left(i\right)}^{1}\in {\mathbb{R}}^{1\times \;65536} \end{array}$$

$F_{B\left(i\right)}^{1}$ represents changing the positions of pixels *F*^1^ in accordance with the chaotic map or permutation order, *B*(*i*) obtained from the sequences of *B*(*n*) with a size of 1 × 65536. Then, the positions of pixels of *F*^2^ are confused using the permutation order or chaotic map, *C*(*i*) obtained from the sequences of *C*(*n*) with a size of 1 × 65536 to obtain the third round or final scrambled image, *F*^3^ with a dimension of 1 × 65536. Then, the final scrambled image is reshaped to 256 × 256 by:


$$\begin{array}{c} F^{3}=\;F_{C\left(i\right)}^{2}\in {\mathbb{R}}^{1\times 65536}\to F^{3}\in {\mathbb{R}}^{256\times \;256} \end{array}$$

The results of the three rounds of confusion for the Pirate image are shown in Figure 5.

**Figure 5:**
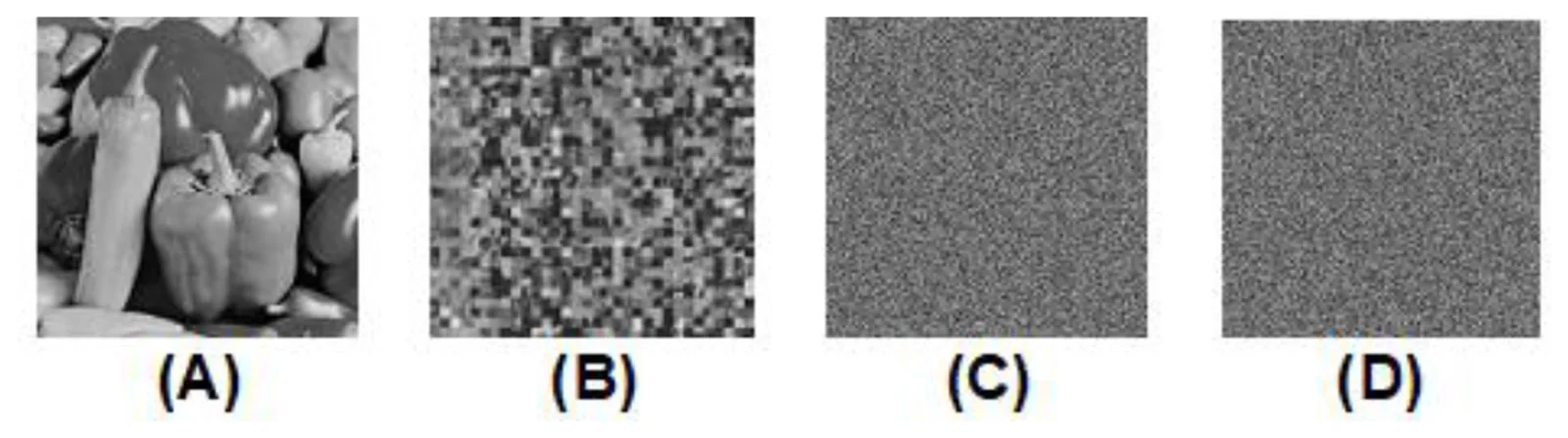
Results of image confusion. **(A)** Plaintext image (256 × 256) (plaintext image reproduced from the USC-SIPI Image Database, USC-SIPI Image Database [2025], https://sipi.usc.edu/database/), **(B)** First round confusion, **(C)** Second round confusion, and **(D)** Third round confusion.

### 3.4 Diffusion

As confusion involves changing the position of the pixels of the plaintext image, it is vulnerable to attacks by intruders. Therefore, it is better to change the pixel values in addition to changing the pixel positions to improve security. In digital logic, *XOR* function has only an inverse. Hence, it plays a vital role in cryptography because of its simple bitwise operation for encrypting data. The encrypted image *E* is directly generated by diffusing *F*^3^ and *K* using represented by Equation 14.


$$\begin{array}{c} E=F^{3}⊕K \end{array}$$ (14)

The scrambled image *F*^3^ is easily retrieved by diffusing the encrypted image, *E*, and the key map (*K*) using the same *XOR* operation.

### 3.5 Decryption

Decryption is the reverse of the encryption process. Therefore, the overall encryption process is reversed to retrieve the plaintext image from the encrypted version. The chaotic maps or permutation orders *C*(*i*), *B*(*i*), and *A*(*i*) are indexed and sorted in ascending order, and their new indices are *C*′(*i*), *B*′(*i*), and *A*′(*i*) respectively. *C*′(*i*), *B*′(*i*), and *A*′(*i*) are called the de-scrambling orders for decryption. The decryption procedure is illustrated by:

(1) *F*^3^ = *E*⊕*K*

(2) *F*^3^∈ℝ^256 × 256^→*F*^3^∈ℝ^1 × 65536^

(3) $F^{2}=\;F_{C^{\prime }\left(i\right)}^{3}\in {\mathbb{R}}^{1\times \;65536}$

(4) $F^{1}=\;F_{B^{\prime }\left(i\right)}^{2}\in {\mathbb{R}}^{1\times \;65536}$

(5) *F*^1^∈ℝ^1 × 65536^→*F*^1^∈ℝ^256 × 256^→*F*^1^∈ℝ^64 × 1024^

(6) $F=F_{A^{\prime }\left(i\right)}^{1}\in {\mathbb{R}}^{64\times 1024}$→*F*∈ℝ^256 × 256^

## 4 Results and discussion

The proposed method is implemented using MATLAB R2023a installed on a personal computer with an Intel^®^ Core^TM^ i5 12th-generation processor and 8 GB RAM. The freely available standard test images from the SIPI image database (USC-SIPI Image Database, 2025 and the Brain tumors 256 × 256 Image Dataset, 2026) were used for experimental analysis. The performance of chaotic map-based image encryption analyzed using various methods is shown in Figure 6. Any image encryption system can be analyzed to test its robustness against various attacks, as shown in Figure 6. The robustness of the system against a brute-force attack can be analyzed using key space and key sensitivity, whereas error can be analyzed using PSNR. The differential attack can be analyzed using NPCR and UACI. The results of the proposed method of encryption are shown in Figure 7. Figures 7A–C are the plaintext image, encrypted image, and the decrypted image with histograms, respectively. The performance of the proposed encryption method is analyzed by histogram, entropy, PSNR, correlation coefficient, and robustness against differential attacks, chosen-plaintext attacks, occlusion attacks, and noise tolerance.

**Figure 6:**
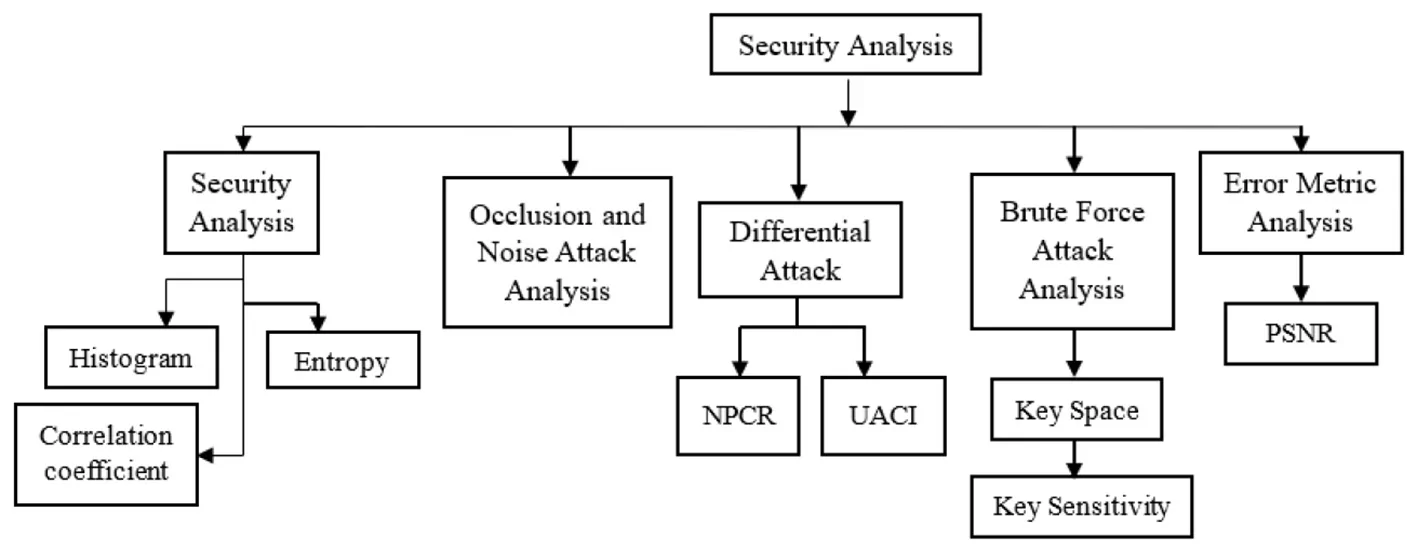
Security analysis of chaos-based image encryption.

**Figure 7:**
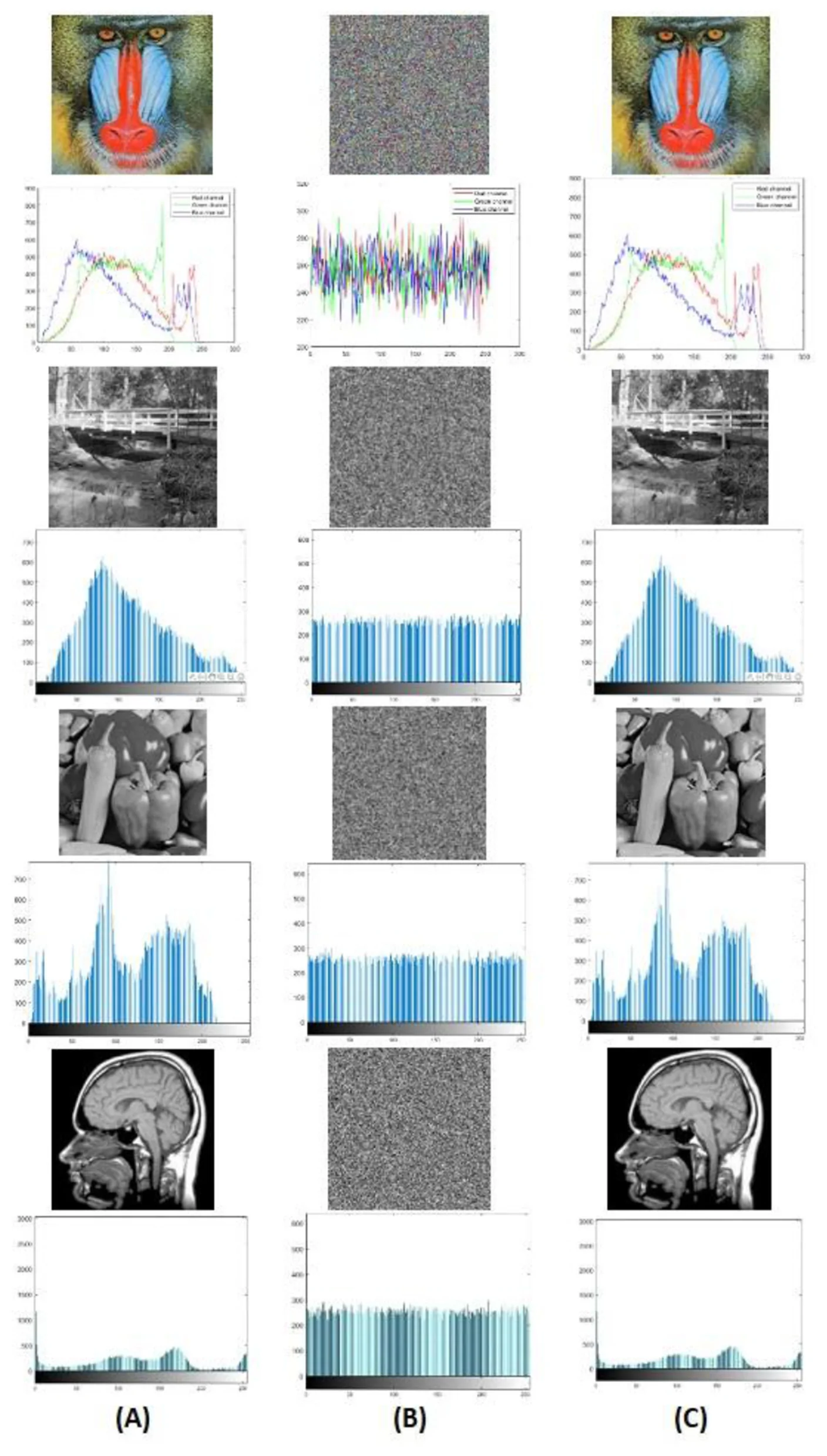
Results of the proposed encryption approach for images of size 256 × 256. **(A)** Plaintext image with histogram (plaintext images reproduced from the USC-SIPI Image Database, USC-SIPI Image Database [2025], https://sipi.usc.edu/database/ and the Brain tumors 256 × 256 Image Dataset, Brain tumors 256 × 256 Image Dataset [2026], https://www.kaggle.com/datasets/thomasdubail/brain-tumors-256x256, licensed under CC0), **(B)** Encrypted image with histogram, and **(C)** Decrypted image with histogram for the Baboon (RGB), Bridge, Peppers, and medical (N_117) images.

### 4.1 Histogram analysis

The histogram is a graphic representation of a 2D discrete dataset to show how the data is distributed. For example, an image is 2D data, and its histogram is drawn between the pixel intensity level on the horizontal axis and the number of pixels on the vertical axis. Hence, the distribution of the histogram components (pixel intensity levels and number of pixels) is used to analyze the performance of the proposed method. In Figure 7, the components of the histogram of the plaintext image are distributed depending on the pixel intensity levels. But in the case of encrypted images, the distribution of the histogram components is almost flat (Singh et al., 2023) and similar for all images. This shows that the proposed encryption method turns a meaningful image into a scrambled image. It indicates that the proposed algorithm provides information security since the randomness of the encrypted image is higher.

### 4.2 Entropy

The measure of uncertainty of information (Singh et al., 2023) of 2D data such as an image is called the entropy (*H*), and it is represented by Equation 15.


$$\begin{array}{c} H=-\sum_{m=1}^{n-1}P\left(m\right)\log_{2}\left(P\left(m\right)\right) \end{array}$$ (15)

where *P*(*m*) is the probability of the corresponding pixel intensity level and *n*−1 is the maximum intensity level (for an 8-bit image, *n* = 256). Usually, the standard entropy value for the encrypted image is close to 8 when there is a high degree of randomness in the image information. The estimated entropy values of the encrypted image in column “b” of Figure 7 are provided in Table 4. The proposed method has an entropy of 7.9974. Hence, the proposed method also has an entropy close to 8, which indicates a high degree of randomness in the information.

**Table 4:** Performance analysis of the proposed encryption method.

Image	NPCR	UACI	Entropy	HCC	VCC	DCC	PSNR	MSE
Baboon grayscale	99.5560	33.4313	7.9974	0.005278	0.004315	0.003018	9.6869	6991.2
Bridge	99.5697	33.5286	7.997	−0.003648	0.001971	−0.001768	8.9245	8388.1
Baboon (RGB)	99.5941	33.5515	7.9974	−0.001957	0.002691	−0.006072	8.9167	8278.9
Peppers	99.6353	33.6352	7.9972	−0.007743	−0.00475	−0.001700	8.8748	8422.3
Pirate	99.5865	33.4824	7.9965	0.004919	0.004212	−0.005819	9.1550	7865.8
N_117	99.6216	33.2196	7.9973	0.010666	0.000218	0.004826	6.9822	14545

### 4.3 Peak signal-to-noise ratio

In the literature, many researchers have used PSNR as a metric for analyzing the differences between the plaintext image and the encrypted image (Gong et al., 2024). PSNR is mathematically represented by Equation 16.


$$\begin{array}{c} PSNR=10\times \log_{10}\left(\frac{P_{\max}^{2}}{MSE}\right)dB \end{array}$$ (16)

$$\begin{array}{c} MSE=\frac{1}{RC}\sum_{i=0}^{R-1}\sum_{j=0}^{C-1}{\left[I\left(i,j\right)-E\left(i,j\right)\right]}^{2} \end{array}$$ (17)

where *R* and *C* are the numbers of rows and columns of the image, *P*_max_ is the maximum intensity level of the 8-bit grayscale of the plaintext image; MSE is used to assess the visual quality of the images. The estimated PSNR and MSE of the test images are provided in Table 4 for the plaintext images and the corresponding encrypted images. For all test images, the MSE is 0 between the plaintext image and the corresponding decrypted image, indicating that each pixel in the reconstructed image exactly matches the corresponding pixel in the plaintext image, which demonstrates perfect reconstruction without loss. From Table 4, it is observed that the proposed method performs well in terms of PSNR and MSE.

### 4.4 Correlation coefficient analysis

The degree of randomness of image information can be analyzed using the correlation (similarity) between adjacent pixels of the image. If the correlation between adjacent pixels of an image is high, then the randomness is low. Hence, the correlation between adjacent pixels of an encrypted image is expected to be close to zero. The correlation coefficient is used in statistics to measure the degree of closeness of two variables (Es-Sabry et al., 2023). So, the correlation coefficient can be used to measure the closeness of adjacent pixels of an image to estimate the degree of randomness of the information in the encrypted image. That is, to measure how the pixels of the encrypted image are correlated with adjacent pixels. The correlation coefficient is calculated directly from either the information or the standard deviation.

The correlation coefficient by the direct method (*r*):

The correlation coefficient can be directly calculated using the values of any two random variables, as shown in Equation 18.


$$r=\frac{n\left(\sum xy\right)-\left(\sum x\right)\left(\sum y\right)}{\left[n\sum x^{2}-{\left(\sum x\right)}^{2}\right]\left[n\sum y^{2}-{\left(\sum y\right)}^{2}\right]}$$ (18)

where *r* represents the correlation coefficient and *n* is the total number of pixels (information), *x* and *y* represent two adjacent pixels of the encrypted image.

Correlation coefficient by Standard Deviation ρ_*xy*_ is given by Equation 19.


$$\begin{array}{c} \rho_{xy}=\frac{cov\left(x,y\right)}{\sqrt{D\left(x\right)D\left(y\right)}} \end{array}$$ (19)

where ρ_*xy*_ represents the correlation coefficient, *cov*(*x, y*) is the covariance between two adjacent pixels, *D*(*x*) and *D*(*y*) represent the standard deviation of pixels at position *x* and *y*, respectively. The covariance and standard deviation are calculated using Equation 20, as given below.


$$\begin{array}{c} cov\left(x,y\right)=\frac{1}{N}\sum_{i=1}^{N}\left(x_{i}-E\left(x\right)\right)\left(y_{i}-E\left(y\right)\right) \end{array}$$ (20)

where

$E\left(x\right)=\frac{1}{N}\overset{N}{\underset{i=1}{\sum }}x_{i}\;$

$E\left(y\right)=\frac{1}{N}\overset{N}{\underset{i=1}{\sum }}y_{i}\;$

$D\left(x\right)=\frac{1}{N}\overset{N}{\underset{i=1}{\sum }}{\left(x_{i}-E\left(x\right)\right)}^{2}\;$

$D\left(y\right)=\frac{1}{N}\overset{N}{\underset{i=1}{\sum }}{\left(y_{i}-E\left(y\right)\right)}^{2}\;$

The Correlation coefficient may be calculated in three ways: (i) Horizontal Correlation Coefficient (HCC), (ii) Vertical Correlation Coefficient (VCC), and (iii) Diagonal Correlation Coefficient (DCC). If the correlation is estimated between pixels of the same row, then it is HCC, whereas if it is estimated between pixels of the same column, then it is VCC. If the correlation coefficient is estimated between diagonally arranged pixels, that is, pixels in different rows and columns, then it is DCC. The correlation coefficient always lies between [−1, 1] to describe the degree of randomness of information. In general, the correlation coefficient is measured between any two variables. If its value is −1, then it indicates a perfect negative correlation between two variables; if its value is 0, then it indicates no correlation between two variables, a correlation coefficient = 1 indicates a perfect positive correlation. In an image encryption system, the correlation coefficient is expected to be close to zero for high randomness of information. In the proposed method, the correlation between adjacent pixels of the encrypted image is close to zero, which indicates low correlation and high randomness. This can be measured using either the Pearson correlation coefficient ρ_*xy*_ or the linear correlation coefficient (*r*).

Table 4 depicts the estimated HCC, VCC, and DCC for the plaintext images and the encrypted images, as shown in Figure 7, using the proposed encryption system. For plaintext images, all three correlation coefficient values are close to 1, which implies poor randomness of image information and hence the images are informative. However, all three correlation coefficient values are close to zero for encrypted images of all plaintext images, and this shows that the degree of randomness is high and the encrypted images are not informative. Hence, the proposed method shows good performance in terms of the correlation coefficient.

### 4.5 Analysis of differential attacks

The differential attack is analyzed based on the differences in pixel intensity values between the plaintext and encrypted images. For this, UACI and NPCR are the two indicators for evaluating image encryption algorithms and their resistance to differential attacks (Mao et al., 2004). NPCR is the difference between two encrypted images obtained with and without a one-bit change of the same plaintext image, and UACI is the mean of the normalized magnitude of the difference between the encrypted image and the plaintext image. Even though these two scores are not suitable as quantitative performance measures of various encryption algorithms, they were used in the literature for quantitative analysis. The mathematical expressions for UACI and represented by Equations 21–23.


$$\begin{array}{c} UACI=\frac{1}{RC}\sum_{i=1}^{R}\sum_{j=1}^{C}\frac{\left|C_{1}\left(i,j\right)-C_{2}\left(i,j\right)\right|}{255}\times 100 \end{array}$$ (21)

$$\begin{array}{c} NPCR=\frac{1}{R\times C}\sum_{i=1}^{R}\sum_{j=1}^{C}D\left(i,j\right)\times 100 \end{array}$$ (22)

$$D\left(i,j\right)=\;\{\begin{array}{c} 1,\;C_{1}\left(i,j\right)=\;C_{2}\left(i,j\right)\\ 0,\;C_{1}\left(i,j\right)\neq \;C_{2}\left(i,j\right) \end{array}$$ (23)

*C*_1_(*i, j*) represents the encrypted image, and *C*_2_(*i, j*) represents the encrypted image obtained by applying the proposed encryption to the plaintext image with a one-bit change or a different key. According to (Mao et al. 2004), NPCR and UACI are above 99 and 33, respectively, and are dependent on the number of cipher rounds and the size of the image. When the number of cipher rounds increases (Chen et al., 2004), the values of UACI and NPCR increase. Therefore, it is concluded that UACI and NPCR may vary with the number of cipher rounds and different encryption algorithms.

The estimated values of NPCR and UACI for the images shown in Figure 7 are given in Table 4. The proposed system yields NPCR and UACI values above 99 and 33, respectively, for all images. This shows that the proposed system achieves a high degree of randomness in the information, which is essential for secure communication. Thus, the proposed method also provides improved encryption for information security.

### 4.6 Occlusion attack

Information in an encrypted image may be damaged either partially or fully by an intruder to make it difficult for users to recover the plaintext image, and this kind of attack is called an occlusion attack. This leads to an inability to recover the information from the original image (Kang and Tao, 2019). The robustness of the proposed method against the occlusion attack is studied using the encrypted Baboon image. For this study, the encrypted image is subjected to cropping by 25%, 50%, and 75%, which leads to damage to the information in the encrypted image and leaves 75%, 50%, and 25% of the information, respectively. It can be easily observed that the information of the plaintext image is recovered from the occluded encrypted image, as the proposed method exhibits strong robustness. The images are cropped by 25%, 50%, and 75%, and the corresponding results are shown in Figure 8. The robustness of the proposed method is quantitatively evaluated using PSNR, and the results are presented in Table 5 for the Baboon image. Table 5 shows that the proposed method exhibits greater robustness against occlusion attacks.

**Figure 8:**
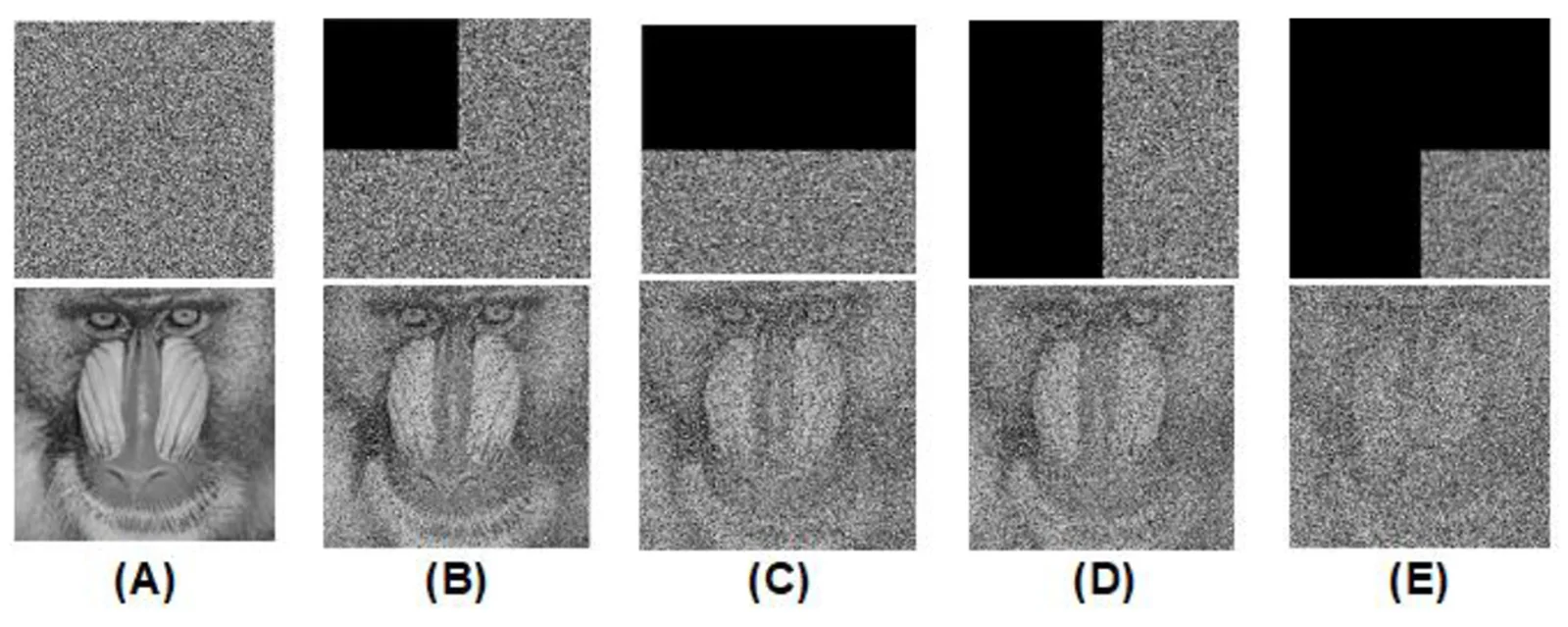
Analysis of the cropping attack on the encryption scheme for the Baboon image (256 × 256). **(A)** No cropping (plaintext image reproduced from the USC-SIPI Image Database, USC-SIPI Image Database [2025], https://sipi.usc.edu/database/), **(B)** 25% cropping, **(C)** 50% cropping from the top, **(D)** 50% cropping from the side, and **(E)** 75% cropping.

**Table 5:** Analysis of the occlusion attack on the Baboon image.

Occlusion level	PSNR
25 % cropping	15.7703
50 % cropping horizontally	12.8048
50 % cropping vertically	12.7407
75 % cropping	11.0159

### 4.7 Analysis of chosen-plaintext attack

To verify the robustness of the proposed system against the chosen-plaintext attack (Diaconu, 2016), cipher-image pixel correlation analysis, cipher difference analysis, and keystream exposure tests are performed. For cipher difference analysis, all-black and all-white images are encrypted using the proposed method, and the difference between the two encrypted images does not show any visible patterns, as shown in Figure 9, which demonstrates the robustness of the proposed system against the chosen-plaintext attack. If correlation exists between adjacent pixels of the encrypted image of either an all-black or an all-white image, then the key map will be exposed (because confusion does not affect an all-black or all-white image), and the system is vulnerable to a chosen-plaintext attack.

**Figure 9:**
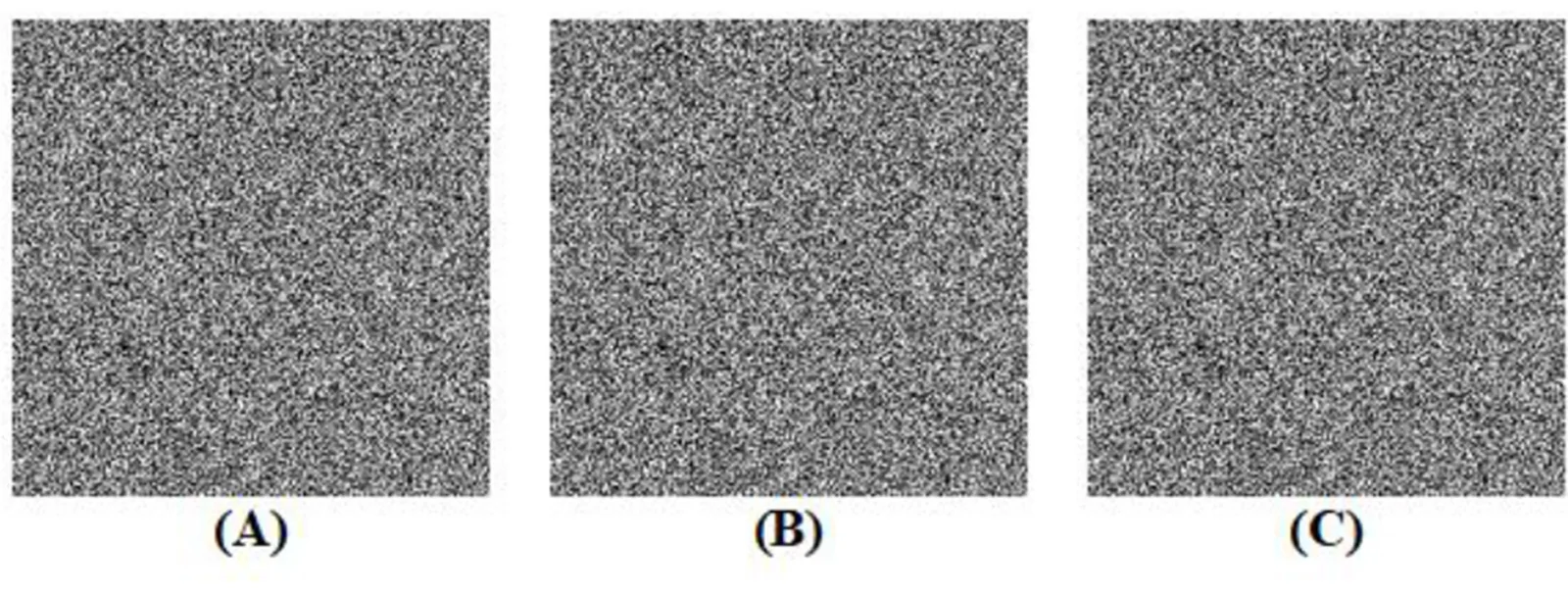
Analysis of the chosen-plaintext attack with an image of size 256 × 256. **(A)** Encrypted all-black image, **(B)** Encrypted all-white image, and **(C)** Difference between the all-black image and the all-white image (no patterns visible).

The pixel correlation of the encrypted image for the all-black image is 0.00113 (nearly 0) for the proposed method, which shows that the system is robust against chosen-plaintext attacks because no correlation exists among pixels. In the keystream exposure test, an all-black image and a random image are generated with the same image size. The two images are encrypted using the proposed system. The *XOR* of the two encrypted images does not expose the keystream.

### 4.8 Analysis of noise tolerance

As with the occlusion attack, the robustness of the proposed system against noise added to the encrypted images is analyzed using the Peppers image. The proposed encryption is applied to the plaintext image, and then 5% and 50% salt-and-pepper noise (Gong et al., 2024) are added to obtain a noisy encrypted image. Then, decryption is applied to retrieve the plaintext image using the proposed method. The results are shown in Figure 10. The proposed system retrieves the plaintext, which indicates that the proposed system is robust against noise even when 50% of the image contains noise.

**Figure 10:**
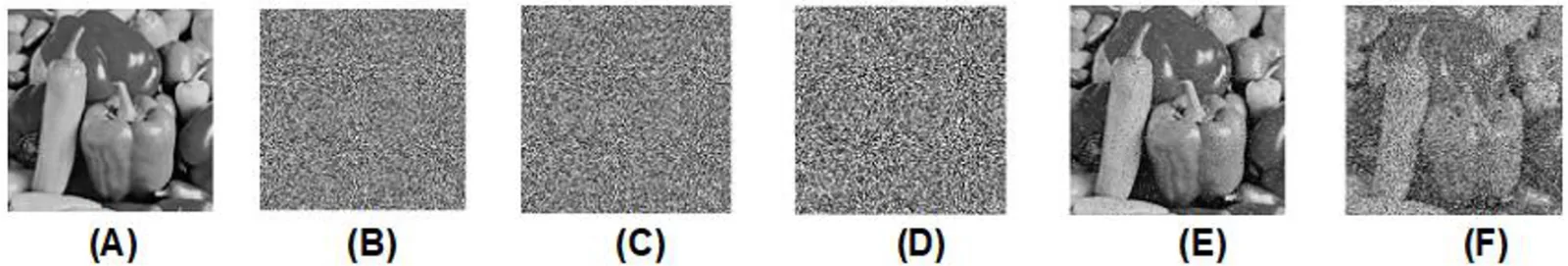
Analysis of the effect of noise on the encrypted image. **(A)** Plaintext image (256 × 256) (plaintext image reproduced from the USC-SIPI Image Database, USC-SIPI Image Database [2025], https://sipi.usc.edu/database/), **(B)** Encrypted image, **(C)** Noise level (5%) of the encrypted image, **(D)** Noise level (50%) of the encrypted image, **(E)** Noise level (5%) of the corresponding decrypted image, and **(F)** Noise level (50%) of the corresponding decrypted image.

### 4.9 Key space analysis

The key image plays a vital role in encryption, as a strong key image enhances the security of the encryption scheme. Key space represents the set of all possible keys that can be used in the encryption scheme to assess its resistance to attacks. As the proposed method uses three initial values and one control parameter, each with a precision of 10^−15^, its key space is 2^199^. Further, the 16 elements of 4 × 4 vector have a precision of 10^−4^, and their key space is 2^212^. Therefore, the total key space is approximately 2^411^. Also, if the elements of the input vector have a precision of 10^−15^, then its key space is 2^797^, and the total key space can also be increased from 2^411^ to 2^996^. However, in this study, the 16 elements of the input vector have a precision of 10^−4^. Hence, the proposed system has strong robustness against brute-force attacks. Also, if there are any small changes in the key (a change in the decimal value of 10^−15^), the generated chaotic map and key map will change, and hence decryption will fail. Therefore, the proposed system has a larger key space and greater key sensitivity and provides strong security. Some comparisons on the key space are given in Table 6.

**Table 6:** Key-space analysis.

Theoretical lower limit	Song et al. (2022)	Alawida (2024)	Alawida and Teh (2026)	Proposed method
2^100^	2^199^	2^156^	2^128^	2^411^

### 4.10 Key sensitivity analysis

The cryptosystem requires a highly sensitive key; even slight changes in the key result in significant changes to the encrypted output. In this article, three initial values of Equation 5 are used as keys. Then the incorrect values of a, b, and c, which are 0.010000000000000100, 0.565000000000000100, and 0.500700000000000101, respectively, are used to decrypt the encrypted image. Figure 11 shows the decrypted images obtained using the correct and incorrect keys. The experimental results clearly demonstrate that the keys are sensitive enough; even a slight change causes decryption to fail. Therefore, the proposed encryption algorithm is highly resistant to brute-force attacks.

**Figure 11:**
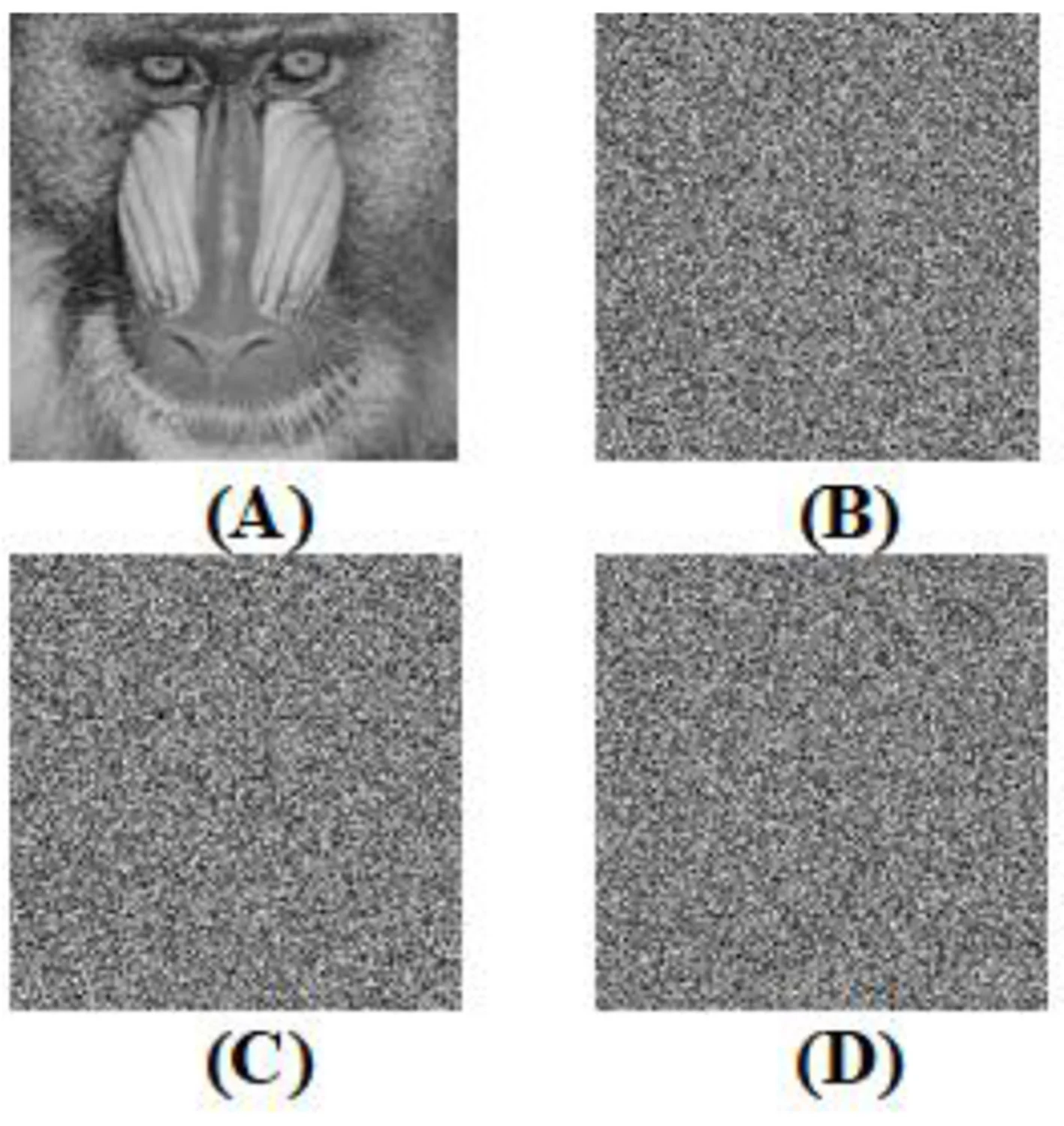
Decryption using correct and incorrect key values (plaintext image reproduced from the USC-SIPI Image Database, USC-SIPI Image Database [2025], https://sipi.usc.edu/database/). **(A)** Decrypted image (256 × 256) with correct keys, **(B)** Decrypted image with incorrect *a*, **(C)** Decrypted image with incorrect *b*, and **(D)** Decrypted image with incorrect *c*.

### 4.11 Runtime analysis

The proposed method is implemented using MATLAB R2023a installed on a personal computer with an Intel^®^ Core^TM^ i5 12th-generation processor and 8 GB RAM. For a plaintext grayscale image of size 256 × 256, the proposed method takes 2.017 s to perform the encryption, and the processing time taken may vary according to the system configuration. The encryption and decryption processing time for the test images is given in Table 7, whereas Table 8 provides a comparison of the time taken by various encryption systems with that of the proposed system. This indicates that the proposed method is highly efficient and feasible.

**Table 7:** Runtime.

Image	Encryption (s)	Decryption (s)
Baboon grayscale	2.017	2.15
Bridge	2.15	2.32
Baboon (RGB)	2.159	2.224
Peppers	2.31	2.678
Pirate	2.20	2.55
N_117	2.535	2.594

**Table 8:** Comparison of running time (256 × 256).

Scheme	Encryption (s)	Decryption (s)
Huang and Gao (2024)	3.3294	2.3673
Xiao et al. (2021)	4.7908	23.7851
Yang et al. (2023)	2.0192	12.2374
Proposed method	2.017	2.15

## 5 Comparative analysis

The performance of the proposed encryption system has been compared with the existing methods, such as those of Liao et al. (2025), Sridharan et al. (2025), Raghuvanshi et al. (2024), Yogi et al. (2025), Ponmahesh and Perumal (2025), and Li et al. (2023) for the Baboon image using NPCR, UACI, entropy, and the correlation coefficient. The values are shown in Tables 9, 10, respectively, for the Baboon image and Peppers image. Also, the proposed method is compared with existing methods, such as those of Song and Zhao (2025), Yogi et al. (2025), Alawida and Teh (2026), Li et al. (2023), Safdar et al. (2025), and Li and Tang (2025) for the Peppers image, and the values are given in Table 10.

**Table 9:** Comparative analysis of the Baboon image.

Architecture/ method	Platform	Key characteristics	NPCR (%)	UACI (%)	Entropy	Correlation coefficient (Encrypted image)		
						HCC	VCC	DCC
Liao et al. 2025) 4D-NDS	MATLAB	High entropy	99.6047	33.5251	7.9993	–	–	–
Sridharan et al. 2025) Quantum scrambling + DNA	IBM Quantum platform	High security of the keys, robust to noise attack	99.59	27.21	7.9878	0.0004	−0.0005	−0.0037
Raghuvanshi et al. 2024) Intertwining logistic map + VGG16	MATLAB	High throughput	99.75	33.39	7.9989	0.0003	0.0024	0.0005
Yogi et al. 2025) Combine Tinkerbell and tent map	Python and Linux-based system	Low power consumption	99.5978	32.7067	7.9994	0.0025	−0.0015	0.0022
Ponmahesh and Perumal 2025) Origami Confusion + Sine Map	Python	Execution time and memory usage	99.618149	33.475238	7.9992	−0.0032	−0.0008	0.0011
Li et al. 2023) Innovative DES structure	MATLAB	High efficiency	99.6120	33.4574	7.9993	0.0059	0.0027	0.0009
Proposed (4D NSCM + Deep network)	MATLAB	Large key space, low runtime, robustness to the chosen attack	99.5560	33.4313	7.9974	0.005278	0.004315	0.003018

**Table 10:** Comparative analysis of the Peppers image.

Architecture/ method	Platform	Key characteristics	NPCR (%)	UACI (%)	Entropy	Correlation coefficient (Encrypted image)		
						HCC	VCC	DCC
Song and Zhao (2025) Integrated maps	Python	Large key space, robustness to noise and cropping	99.60	33.48	7.993	−0.000107	0.006354	0.020277
Alawida and Teh (2026) 3D S-boxes	IoT SCS device	Minimal computational complexity	99.6645	33.4456	7.9394	0.0031	0.000321	0.00011
Yogi et al. (2025) Combination of Tinkerbell and tent maps	Python and Linux-based system	Low power consumption	99.5895	32.6052	7.9977	0.0010	−0.0013	0.0017
Li et al. (2023) Innovative DES structure	MATLAB	High efficiency	99.6040	33.4861	7.9993	0.0194	−0.0013	0.0010
Safdar et al. (2025) S-Box + DNA	–	Minimal computational complexity	99.6155	28.95	7.9967	0.0033	−0.0012	0.0002
Li and Tang (2025) 2D Quantum	IBM Quantum platform	High key space, high key sensitivity, and low adjacent pixel correlation	99.6018	33.4689	7.9993	0.0002	0.0005	0.0014
Proposed (4D NSCM + Deep network)	MATLAB	Large key space, low runtime, and robustness to the chosen attack	99.6353	33.6352	7.9972	−0.007743	−0.00475	−0.001700

From Tables 9, 10, it is observed that the proposed system achieves NPCR values of 99.5560 and 99.6353 for the Baboon and Peppers images, respectively, which indicate good encryption. UACI values are 33.4313 and 33.4824 for the Baboon and Peppers images, respectively, which also indicate good encryption. Also, the proposed system performs well in terms of entropy and the correlation coefficient. The experimental results of the proposed method show that it also provides strong security for image information against various attacks. The new key map generation method improves the key complexity, and the modification of the monotonic function to a 4D chaotic map for confusion and the key map for diffusion further improves the chaotic nature of the information and converts the meaningful image into a scrambled image.

## 6 Conclusion

Thus, a new 4D chaotic system integrated with a deep network is introduced for image encryption. The block-wise and pixel-wise confusion based on the proposed chaotic map provides greater randomness in the information. The generation of the key image using a deep network not only improves the security of image information but also enhances the key space of the encryption system. Also, the proposed method has greater key sensitivity due to the use of a very small input vector as the initial values to generate the key image. Finally, the experimental results demonstrate that the proposed encryption system improves the randomness of image information, as measured by the image histogram, entropy, the correlation coefficient, NPCR, and UACI.

## Data Availability

The original contributions presented in the study are included in the article/supplementary material, further inquiries can be directed to the corresponding author/s.
